# Chemical Element Profiling in the Sera and Brain of Bipolar Disorders Patients and Healthy Controls

**DOI:** 10.3390/ijms232214362

**Published:** 2022-11-18

**Authors:** Vishnu Priya Sampath, Shiv Vardan Singh, Ilana Pelov, Ofir Tirosh, Yigal Erel, David Lichtstein

**Affiliations:** 1 Department of Medical Neurobiology, Institute for Medical Research Israel-Canada, Faculty of Medicine, The Hebrew University of Jerusalem, Jerusalem 91905, Israel; 2 Jerusalem Mental Health Center, Eitanim Psychiatric Hospital, Jerusalem 91060, Israel; 3 The Fredy and Nadine Herrmann Institute of Earth Sciences, Faculty of Science, The Hebrew University of Jerusalem, Jerusalem 91904, Israel

**Keywords:** bipolar disorder, vanadium, boron, aluminum, chemical elements, brain, serum

## Abstract

Bipolar Disorder (BD) is a severe recurrent affective mood disorder characterized by a wide range of lifelong mood swings, varying between depressive and manic states. BD affects more than 1% of the world’s population irrespective of nationality, ethnic origin, or socioeconomic status and is one of the main causes of disability among young people, leading to cognitive and functional impairment and raised mortality, particularly death by suicide. Trace elements play a vital role in many biochemical and physiological processes. Compelling evidence shows that element toxicity might play a crucial role in the onset and progression of neurodegenerative disorders, but their involvement in mood disorders has been scarcely studied. In the present investigation, we determined the concentration of 26 elements in the serum of BD patients before and after treatment and in postmortem brain samples from BD patients and compared them with matched controls. The only element that was reduced significantly in the serum following treatment was vanadium (V). Furthermore, the concentration of Al, B, Cu, K, Mg and V were significantly lower in the pre-frontal cortex of BD patients compared with those of the controls. A comparison of Spearman’s rank correlation coefficients between the elements in the serum and brain of BD patients and control groups pointed to boron and aluminum as being involved in the disease. These results suggest that there is a disturbance in the elements’ homeostasis and the inter-elements’ relationship in the brain of BD patients and advocate a thorough examination of the possible involvement of chemical elements in different stages of the disease.

## 1. Introduction

Trace elements play a vital role in many biochemical and physiological processes, affecting various biochemical reactions by acting as cofactors for many enzymes, as well as by stabilizing enzymes and protein structures [1,2]. The homeostasis of trace elements in the extracellular space relies on the processes of absorption, distribution, biotransformation, and excretion [3]. Their balance within the brain is regulated in a complex manner by brain barrier systems such as the blood–brain barrier (BBB) and the blood–cerebrospinal fluid (CSF) barrier [4,5]. On the other hand, excess amounts of elements may induce cellular damage resulting in a variety of syndromes caused by abnormal proteins synthesis and folding, lipid peroxidation and oxidation of ROS-scavenging products [3]. Convincing evidence shows that element toxicity might play a crucial role in the onset and progression of neurodegenerative disorders such as Parkinson’s and Alzheimer’s diseases [6,7,8], but their involvement in mood disorders has been studied rarely [9,10].

Bipolar Disorder (BD) is a severe recurrent and affective mood disorder characterized by a wide range of lifelong mood changes, varying between depressive and manic states [11,12]. It affects more than 1% of the world’s population irrespective of nationality, ethnic origin, or socioeconomic status. BD is one of the main causes of disability among young people, leading to cognitive and functional impairment and increased mortality, particularly death by suicide [11]. A large number of genes have been suggested to be involved in the disease, and environmental factors such as stress, are considered important as well [13,14,15].

The possible involvement of trace elements in the etiology of BD was previously addressed and some studies determined their levels in the serum and brain of BD patients. One study found increased lead (Pb) and cadmium (Cd) levels in both the blood and urine of BD patients [9] and the authors suggested that BD may reflect a past high lead exposure in childhood and adolescence. Other have shown that serum selenium (Se) and zinc (Zn) concentrations were significantly lower in BD patients than those in the healthy controls [16,17,18]. The study by Dean et al. [10], to the best of our knowledge, is the only one in which trace elements were determined in the brain tissue of BD patients, and detected lower levels of strontium (Sr) in the brain of BD patients compared with those in the controls.

In recent years, we and others provided strong evidence for the involvement of the plasma membrane transporter Na^+^, K^+^-ATPase and its inhibitors, endogenous cardiac steroids such as ouabain, in BD [19,20,21,22,23]. As many elements interact with and inhibit the activity of Na^+^, K^+^-ATPase [24,25,26], we hypothesize that alterations in the serum and brain level of these elements may be involved in the etiology of BD. To address this conjecture, element concentrations were determined before and after treatment in the serum of BD patients experiencing manic symptoms and in the postmortem prefrontal cortex (PFC) of BD patients and matched controls. We conducted a thorough inter-elemental correlation analyses between all the tested element pairs in the serum and brain samples, which led to the identification of boron (B) and aluminum (Al) in the sera and brain, respectively, as elements the correlations of which with other elements are most significantly altered in BD.

## 2. Results

### 2.1. Chemical Elements in the Sera of BD Patients before and after Treatment

Considering the hypothesis that various elements are involved in the etiology of BD, and reported differences in chemical element concentrations in the sera of BD patients, we first addressed the possibility that element concentrations are altered following treatment. Blood samples were collected from 11 patients in the morning, immediately upon their admission to the acute psychiatric units of Eitanim Psychiatric Hospital (Eitanim, Israel) due to a manic psychotic episode. All the patients were females, average age 35.36 ± 12.96 (S.D) years and were treated initially with Clothiapine, clonazepam or lorazepam or a combination of these drugs, and added mood stabilizers such as lithium and/or antipsychotic drugs after the first blood sampling. The patients received a standard hospital diet without any supplementation that may contain high metal levels. To the best of our knowledge, none of the patients were exposed to metals in the course of their work routine or during the hospitalization. A second blood sample was collected 2–3 weeks later, after the patient had stabilized and been released from the acute inpatient unit or the hospital. Mood and behavior stabilization, manifested by longer sleep periods, reduced grandiose delusions, irritability and talkativeness, was determined by the professional medical staff. The samples were extracted, and elemental concentrations were determined, as described in Materials and Methods. Elemental concentrations in the sera of the patients, before and after treatment are tabulated in Table 1. As the data does not have a normal distribution and the sample size was small (11 pairs), a nonparametric test, the exact one-tailed Wilcoxon matched-pairs signed-ranks test was used to evaluate the effect of the treatment. Of the 20 elements measured, (the remaining six were below the limits of detection), only the concentration of V changed significantly following treatment (*p* = 0.034). This finding focused our attention on V in subsequent analyses and experiments and these are presented elsewhere [27].

Analysis of the correlation of the element concentrations in the serum with age revealed a lack of correlation for almost all the elements. Statistically significant negative correlations for Ca^++^ and SO_4_ before treatment and a positive correlation for Mg^++^, after treatment, were detected (Table 2).

### 2.2. Element Concentrations in the PFC of BD Patients and Matched Controls

Freshly frozen PFC tissues were obtained from the human postmortem brain tissue collection at the Human Brain Collection Core, National Institute of Mental Health (NIMH). Demographic characteristics of the control and BD patient brain samples, gender, cause of death, use of alcohol, mood stabilizers and psychoactive drugs, are summarized in Table 3. According to the analysis of variance (ANOVA), the groups did not differ in postmortem interval, brain weight and pH. Chi square analysis indicated that the groups did not differ in terms of gender. Most of the studied BD patients used psychoactive drugs and all of them committed suicide, whereas only 25% of the control group used psychoactive drugs and none of them committed suicide.

**Table 3:** Characteristics of BD patients and unaffected controls.

	Control (20)	Bipolar Patients (20)
Age at death (years)	43.12 ± 3.21	42.25 ± 3.23
Cause of death (Suicide/other)	0/20	15/5
Brain weight (g)	1382.75 ± 23.08	1511.75 ± 37.58
Postmortem interval (h)	30.70 ± 3.03	28.97 ± 3.94
Brain tissue pH	6.44 ± 0.05	6.34 ± 0.05
Sex (Men/Women)	14/6	14/6
Alcohol history (none/positive)	4/16	5/15
Use of Mood stabilizers (none/positive)	0/20	8/12
Use of Psychoactive drugs (none/positive)	14/6	0/20

A total of 5–10 mg wet brain tissue samples were extracted, and element concentrations were determined, as described in the materials and methods. The results are shown in Table 4.

A comparison of element concentrations between the control and BD groups revealed a statistically significant lower concentrations of six elements (Al, B, Cu, K, Mg and V) in the BD brain samples. No significant differences were observed for the remaining elements analyzed.

Analysis of elemental concentrations in all brain samples as a function of age did not reveal any significant correlation (Table 5).

### 2.3. Inter-Element Correlations in Serum Samples

To bring out the inter-relationship between the determined elements, element–element correlations were determined. The Spearman correlation was used to calculate the correlation coefficients (r_s_). The use of the Spearman coefficient was chosen because of the small sample sizes (as low as 8) and the uncertain metric of the variables. The r_s_ between all sample pairs of the 20 elements, taken upon patient admission and those obtained after treatment were computed.

As shown in Table 6, Tables S1 and S2, 28 correlations between sera elemental concentrations were found in the samples before treatment and only 16 were detected after treatment.

**Figure 1:**
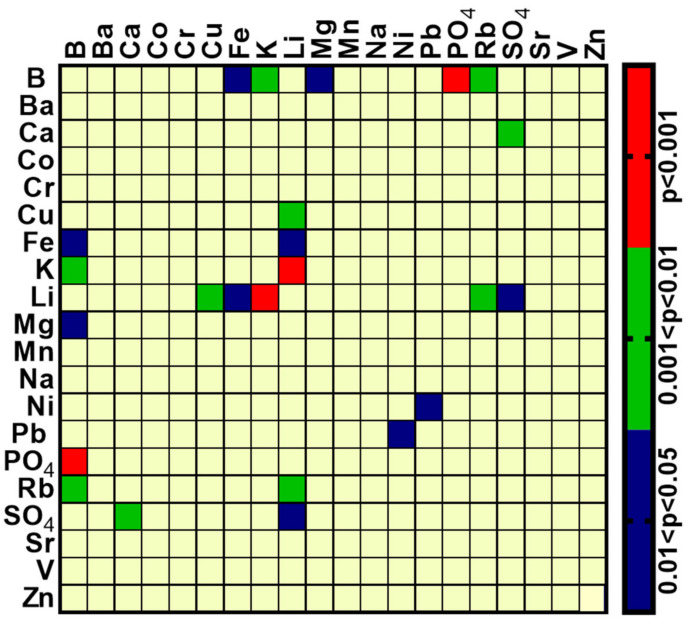
Heat map of the comparison of Spearman correlation coefficients (rs) of element concentrations between serum samples of BD patients before and after treatment. The rs were computed for all pairwise comparisons, separately for the samples before and after treatment. The two-tail statistical significance of the difference between the rs of the two groups for each pair of elements was tested, as described in the results section; only those elements statistically different are shown, grouped according to the scale to the right.

To ascertain whether the difference was genuine or merely a random fluctuation, further analysis is required. The two coefficients (before and after treatment on the same individuals) were compared by using a special adaptation of Fisher’s *z*-transformation [28] that can be assumed to be normally distributed in such cases [29] and is suitable for Spearman coefficients [30,31]. The two-sided *p*-values that resulted from the comparisons of all 190 pairs of elements were grouped into four categories: *p* < 0.001; 0.001 < *p* < 0.01; 0.01 < *p* < 0.05; *p* > 0.05, and these were plotted in the form of a standard categorical heat map by using GraphPad Prism v 8.4.2 (GraphPad Software, Inc., Sad Diego, CA, USA) and shown in Figure 1. As can be seen, 12 r_s_ differences were found at *p* < 0.05, while the number expected by chance alone is 9–10. The two elements that showed the highest number of differences at *p* < 0.01 and *p* < 0.001 were Li and B. Interpretations of this analysis are further addressed in the discussion.

### 2.4. Inter-Element Correlations in Brain Samples from BD and Matched Control Samples

Spearman’s rank correlations were computed between element-pairs. As in the serum analyses, the Spearmen coefficient was chosen because of the small sample sizes in each group (as low as 8 in the BD patients, as low as 11 in the controls) and the uncertain metric of the variables. Whereas 77 correlations (all positive) were observed in the control samples, only 35 (29 positive and 6 negative) were detected in the BD patients’ samples (Table 7, Tables S3 and S4, see Supplementary Materials). Strong positive correlations (r_s_ above 0.7) were found in the 26 control samples, only six were found in the BD samples. Several elements that correlated with numerous other elements in the control group, did not correlate with most of these in the BD group. For example, Al correlated with eleven elements in the control group (B, Ba, Ca, Co, Cr, Li, Ni, Pb Sr, U, V), but only with one element (V) in the BD group; Ba correlated with eight elements in the control group (Co, Cr, Fe, Li, Mn, Sr, U, V) versus only with three in the BD group (Ca, Sr, U) and Ca correlated with nine elements in the control group (Cd, Co, Cr, Ni, Pb, Sr, U, V, Zn), versus only with 4 (Ni, Pb, Sr, U) in the BD group (Table 7).

**Figure 2:**
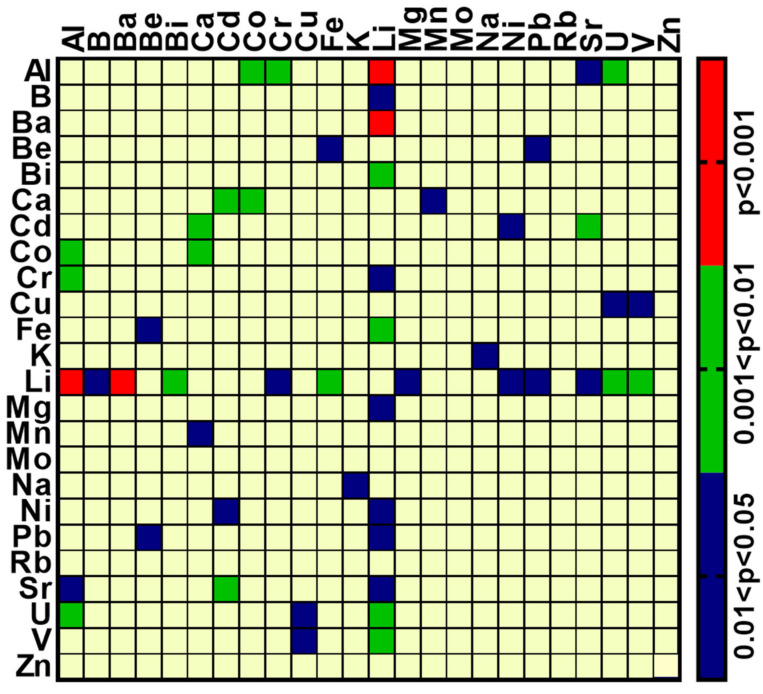
Heat map of the comparison of Spearman correlation coefficients (rs) of element concentrations between control and BD patients. The rs were computed for all pairwise comparisons, separately for the control and the BD patients. The two-tail significance of the difference between the rs of the control and BD groups for each pair of elements was tested, as described in the results section; only those elements statistically different are shown, grouped according to the scale to the right.

In an attempt to explore the elements involved in BD and the apparent large differences between the BD and control groups, we compared the latter’s Spearman correlation coefficients. To this end, Fisher’s z-transformation was used to convert all the computed coefficients to variables that can be assumed to be normally distributed [30] and each such control group variable was then compared with the corresponding variable from the BD group according to a simple two-tail z-test. The *p*-values were grouped into four categories: *p* < 0.001; 0.001 < *p* < 0.01; 0.01 < *p* < 0.05; *p* > 0.05, and these were plotted in the form of a standard categorical heat map (GraphPad Prism v 8.4.2 (GraphPad Software, Inc.) (Figure 2).

Of a total 76 coefficients, 26 were reduced significantly in BD patients, and none showed any significant increase. The 26 included the correlation of Li with Al, B, Ba, Bi, Cr, Fe, Mg, Ni, Pb, Sr, U and V; Al with Co, Cr, Sr and U; Be with Fe and Pb; Ca with Cd, Co, and Mn, and Cd with Ni and Sr. Interpretations of this analysis are further addressed in the discussion.

## 3. Discussion

The pathophysiology of BD remains to be elucidated and there are no diagnostic or prognostic biomarkers for the condition. This study addresses an intriguing hypothesis that exposure to environmental chemical elements may play a role in BD. An indication of the possible importance of elements in BD is the wide, long-lasting, use of Li for preventing the disorder’s manic and depressive recurrences [32]. Previous studies presented elemental variations in the serum [9,16,17,18,33] and brain [10] of BD patients compared with those in the controls and a large body of evidence, cited in the introduction, from studies performed 40 years ago, implicated V in the illness. In the present study, we determined chemical elements in the serum of BD patients, before and after treatment, and also compared their concentrations in the PFC of BD patients with those in the matched controls.

### 3.1. Alterations in Element Concentrations in the Serum and Brain

A hypothesis regarding the involvement of V in the etiology of BD was put forward about 40 years ago [34,35]. In the present study, serum V was the only element that was reduced following treatment of BD patients (Table 1) and lower concentrations of V were detected in the brain of such patients (Table 4). These results prompted us to investigate the effects of this metal on behavior and related molecular alterations in mice brains. The results of these experiments, which advocate revival of the hypothesis of V involvement in BD, are presented elsewhere (Sampath et al., adjacent publication).

Zn, Mg and Cu were suggested to be involved in depressive disorders and proposed as a potential clinical marker for these conditions [36,37,38]. This was based on many reports indicating significant changes in blood levels of these elements during a depressive episode [18,39,40]. Our results do not support this notion, as the serum concentration of these elements did not change following treatment (Table 1). However, in our study, element levels were determined 2–3 weeks after treatment and changes in the levels at a longer treatment time cannot be excluded.

The correlation of serum elemental concentrations with age (Table 2) showed a significant negative correlation for Ca^++^ and SO_4_, phenomena described previously [41,42].

We found that the concentrations of six elements (Al, B, Cu, K, Mg and V) were significantly lower in the brain of BD patients compared with those of the control (Table 5). Overall, there was good agreement with previously published values for individual elements [3], although our determinations yielded significantly lower values for some elements, including Co, Cu, and K. To the best of our knowledge, the study by Dean et al. [10] is the only previous study that compared brain elemental concentrations between patients with mood disorders and healthy controls. Our study found lower concentrations in cortical Sr, Cd and Ru in samples from patients with major depressive disorder, as well as lower concentrations of Sr in patients with BD. The reason for the lower concentrations of some elements in our and Dean’s study is not known. It is noteworthy that the element balance within the brain is regulated in a complex manner by brain barrier systems [5] and their homeostasis relies on the processes of absorption, distribution, biotransformation, and excretion [4]. Hence, alteration of one of these processes, due to genetics, food intake or environmental causes, may be the underlying mechanism for the reduced elemental concentrations in BD and other mood disorders.

The correlation of the elemental concentrations in all the brain samples as a function of age did not reveal any significant correlation (Table 6). This concurs with a recent study showing that the concentrations of nine elements in post-mortem Alzheimer’s patients did not correlate with age [43].

### 3.2. Alterations in Inter-Elemental Correlations in Serum and Brain

It bears mentioning that the correlations between different chemical elements cannot be explained definitively by any single process, but rather reflect a very complicated system of biochemical reactions and might be just a concomitant phenomenon.

Most of the differences in the inter-element correlations between serum samples before and after treatment were of Li and B. As 4 of the 11 patients received Li as a part of their treatment, a relatively large number of differences of this element between samples before and after treatment is expected. The number of differences for B is surprising and highly significant. At *p* < 0.01, 3 r_s_ differences were found for B (Figure 1) although the probability of an element showing 3 or more differences by chance is 1.4%. Furthermore, at *p* < 0.001, B was the only element with a difference of r_s_ when the probability of an element to have 1 or more differences by chance is 1.5 × 10^−5^. Notably, B is one of the elements that was significantly reduced in the BD brain (Table 4). Although, at present, we do not understand the reason for the change in correlations for B following treatment, this analysis focuses attention on this element. Boron is used by plants and humans as a microelement and has been reported to be a metal chelator with antioxidant, non-cytotoxic, anti-genotoxic and anti-carcinogenic characteristics [44,45]. In the brain, B is necessary for neural activity and boron deficiency leading to defects in brain electrical activity, movement as well as loss of consciousness and psycho-motor activity, and short-term memory weakness [46]. Any association of B with BD is, to the best of our knowledge, not mentioned in the literature.

We determined inter-elemental correlations in the BD and control brain groups (Table 7,  Tables S1 and S2). Our findings, reflected by inter-elemental relationships, suggest that there is an imbalance in macro and trace element homeostasis in the BD brain compared with that in psychiatrically healthy individuals. The large number of differences found between BD and control samples supports the notion, which remains to be tested, that common entities which affect elemental concentrations, such as the levels of metal binding proteins and metal transporters, are altered in BD.

A comparison of the Spearman correlation coefficients from the BD and control brain samples revealed that 26 (out of a total 276) were significantly reduced in samples from BD patients compared with those of the controls (Figure 2). However, as many of the BD patients were treated with Li (Table 4), the r_s_ between BD and the control groups for this element should be ignored, as most likely it is attributable to the treatment and not to the disease. Excluding the correlations with Li, there were 253 total correlations, 14 of them significantly reduced in BD. Assuming a random event, one would expect about 13 correlations at the level of *p* < 0.05. This suggests that there are very few, if any, significant correlations between the elements in BD and they are probably not involved in the disease. An exception is Al. This element is significantly correlated in BD samples with four elements (Co, Cr, Sr and U, Figure 2), whereas only one could be expected by chance. Interestingly, Al was one of the elements that had significantly lower concentrations in the brain of BD patients as compared with those in the controls (Table 5). This implies that brain Al may be a factor that either participates in the etiology of BD or is altered in the patient’s brain during the disease or by the treatment. Aluminum is the third most common element on the Earth’s surface, after oxygen and silicon, representing approximately 8% of the Earth’s crust by weight. In the body, Al accumulates mainly in the bones (50%), lungs (25%), kidney and liver and enters the brain primarily through the BBB [47]. Various studies demonstrated that ingestion and exposure to high aluminum levels can result in serious health problems [48]. Although numerous investigations explored the possible involvement of Al in Alzheimer’s and other neurodegenerative diseases [49,50] our study is, to the best of our knowledge, the first demonstration of its possible involvement in BD.

Cumulatively, we revealed several alterations in chemical elements in the serum and brain associated with BD: a reduction in the serum V concentration in BD patients following treatment; lower levels of Al, B, Cu, K, Mg and V in the PFC of BD patients compared with those in matched controls; a difference in the number of inter-element correlations in the serum before and after treatment with B, the most significantly altered element; a difference in the number of inter-element correlations in the PFC of BD patients compared with those in controls, with Al the most significantly altered element. These results support the hypothesis that an imbalance in element homeostasis may play a role in BD and advocate an in-depth investigation of this conjecture.

## 4. Materials and Methods

### 4.1. Serum from BD Patients

The study on human serum samples was approved by the Helsinki Committees of Eitanim and Hadassah Hospitals, Jerusalem (Approval number 1-18). We recruited patients over 18 years of age, who had developed severe manic symptoms and were admitted to the Emergency Room at the Eitanim Psychiatric Hospital (Eitanim, Israel). A total 11 female patients were recruited between 2018 and 2019. Diagnosis of a manic episode was made by the treating psychiatrists and verified by a staff psychiatrist from the research team. Blood samples (10 mL) were collected from the patients immediately upon admission. A second blood sample (10 mL) was collected 2–3 weeks later following initial stabilization and partial remission of the symptoms, which enabled the patients to give written informed consent for participation in the study. First samples from patients who did not consent at any time to participate in the study were discarded. Following centrifugation (4 °C, 2000× *g*, 15 min) of the samples, the sera were frozen and stored at −80 °C until use.

### 4.2. Brain Postmortem Samples

All postmortem human brain tissue samples used in this study were obtained from the Human Brain Collection Core (HBCC), Intramural Research Program, of the NIMH, NIH (http://www.nimh.nih.gov/hbccm, accessed on 28 November 2019), Bethesda, MD, USA. Samples from 20 BD patients and 20 controls were analyzed. The demographic and clinical characteristics of this cohort are presented in Table 3. The NIMH received ethics approval for brain collection.

### 4.3. Determination of Elements in Serum and Brain Tissue

Sample preparation for elemental analysis was carried out in a clean laboratory in the Institute of Earth Sciences at the Hebrew University. The clean laboratory includes a monitored positive pressure air supply with HEPA filtration and entirely non-metallic construction. A total of 5–10 mg wet brain tissue and 5 mL sera were extracted in pre-conditioned PFA. Teflon beakers containing high purity nitric acid (HNO_3_) and hydrogen peroxide (H_2_O_2_) (2 mL 70% HNO_3_ + 1 mL 30% H_2_O_2_). After complete dissolution, the samples were dried almost completely, re-dissolved in 1% high purity HNO_3_, and diluted in triple distilled water (18Ω) to the desired volume for analysis. The concentration of 26 elements (Li, Na, K, Rb, Be, Mg, Ca, Sr, Ba, V, Cr, Mn, Fe, Co, Ni, Cu, Zn, Mo, Cd, B, Al, Pb, Bi, U, P (as PO_4_), S (as SO_4_)) were determined with Inductively Coupled Plasma Mass Spectrometry (ICP-MS) (Agilent 7500cx and Agilent 8900). The ICP-MS was calibrated with a series of multi-element standard solutions (Merck ME VI) and a blank (triple distilled water). All elements were measured using collision cell (He at 5 mL/min). A solution of internal standards (50 μg/L Sc, 5 μg/L Re and 5 μg/L Rh) was injected alongside the samples during the analytical session for drift correction. The USGS SRM’s (T-235, T-221; in dilute HNO3 matrix) were examined after calibration for accuracy assessment. Precision, determined by multiple runs of 1–2 standards, was estimated as <5%. The results are expressed as the mean ± SE of 20 brain samples or 11 serum samples.

### 4.4. Statistical Analyses

The demographic and clinical characteristics of the post-mortem brain samples from control and BD subjects were analyzed with two-way analysis of variance (ANOVA). Chi-square analysis was used to detect gender differences. The nonparametric Exact One-tailed Wilcoxon matched-pairs signed ranks test was used to evaluate the differences between pairs. Element concentrations in the brain are expressed as the median and the exact one-tailed Mann–Whitney test was used. The nonparametric two-tailed Spearman’s Rank Correlation test was used to evaluate inter-element association between element concentration and age and element correlations among themselves. Comparisons between two correlation coefficients were made with a special adaptation of Fisher’s *z*-transformation [28] that can be assumed to be normally distributed in such cases [29] and which is suitable for Spearman coefficients [30,31]. All analyses were performed with GraphPad Prism v 7.03 (GraphPad Software, Inc.), *p* < 0.05 was considered significant.

## Figures and Tables

**Table 1:** Elemental concentrations in sera of BD patients before and after treatment.

Element	Element Concentration						
	Before Treatment	SD	After Treatment	SD	Sum of Positive Ranks	Sum of Negative Ranks	Statistics *p*
B (µg/L)	58	36	63	59	39	−27	0.311
Ba (µg/L)	2.73	0.98	2.92	0.83	28.5	−26.5	0.469
Ca (mg/L)	136	8.49	134	5.06	22	−33	0.303
Co (µg/L)	0.65	0.32	0.63	0.34	12.5	−15.5	0.492
Cr (µg/L)	6.01	4.73	3.93	2.20	17	−49	0.087
Cu (mg/L)	1.61	0.17	1.51	0.25	6.5	−29.5	0.054
Fe (mg/L)	6.6	5.5	14	24.83	29	−26	0.460
K (mg/L)	764	479	898	562.4	35	−31	0.449
Li (mg/L)	22	9.3	18	4.48	23.5	−42.5	0.215
Mg (mg/L)	33	3.49	33	4.38	30.5	−35.5	0.429
Mn (µg/L)	5.91	8.25	2.24	1.28	26	−40	0.287
Na (%)	0.43	0.02	0.42	0.03	13.5	−41.5	0.085
Ni (µg/L)	6.69	7.79	4.57	2.35	28	−27	0.490
Pb (µg/L)	0.60	0.55	0.76	0.66	25	−11	0.203
PO_4_ (mg/L)	718	310	794	288.2	44	−22	0.182
Rb (mg/L)	0.77	0.49	0.89	0.54	34	−32	0.482
SO_4_ (%)	0.50	0.05	0.49	0.03	20.5	−34.5	0.254
Sr (µg/L)	47	11.22	49	11.29	36	−9	0.062
V (µg/L)	1.69	2.46	0.63	0.36	9	−46	0.034 *
Zn (mg/L)	1.22	0.28	1.40	0.90	29.5	−36.5	0.389

Metals were extracted and determined in sera samples (*n* = 11 except B and Pb in which *n* = 8 and 9, respectively), as described in the materials and methods. * The effect of the treatment was analyzed utilizing nonparametric Exact One-tail Wilcoxon Signed-Ranks Test.

**Table 2:** Spearman’s rank correlations coefficient between sera elemental concentrations and age before and after treatment.

Element	Before Treatment	After Treatment
B (μg/L)	−0.3196	−0.1383
Ba (μg/L)	−0.0804	0.2112
Ca (mg/L)	−0.6870 *	−0.0848
Co (μg/L)	−0.1552	-0.2953
Cr (μg/L)	−0.0206	0.0183
Cu (mg/L)	−0.4792	−0.1528
Fe (mg/L)	−0.1690	0.4110
K (mg/L)	−0.0938	0.4749
Li (mg/L)	−0.1287	−0.1190
Mg (mg/L)	−0.1759	0.7425 *
Mn (μg/L)	0.0756	0.2425
Na (%)	−0.1721	−0.2910
Ni (μg/L)	0.0655	−0.1432
Pb (μg/L)	0.3569	0.1212
PO_4_ (mg/L)	−0.5525	0.5845
Rb (mg/L)	0.0823	0.5332
SO_4_ (%)	−0.7875 **	−0.0277
Sr (μg/L)	−0.3836	−0.0274
V (μg/L)	0.0742	0.2268
Zn (mg/L)	−0.0206	0.2151

Spearman’s rank correlations coefficient between sera elemental concentrations and age before and after treatment were calculated using GraphPad Prism v 7.03 Significantly different from samples before treatment, *p* value: 0.033 (*), 0.002 (**).

**Table 4:** Elemental concentrations in brain samples of BD and psychiatrically healthy individuals.

Metal	Control					Bipolar					Statistics *p*
	Mean (*n*)		SD	Median	Sum of Rank	Mean	(*n*)	SD	Median	Sum of Rank	
Al (μg/g)	10.91	19	20.29	1.84	443	1.95	20	2.91	0.93	337	0.039 *
B (ng/g)	0.66	20	0.32	0.65	486	0.45	19	0.32	0.42	294	0.007 *
Ba (ng/g)	0.09	20	0.09	0.05	410	0.07	20	0.06	0.06	410	0.500
Be (μg/g)	0.003	11	0.002	0.003	116	0.003	08	0.001	0.002	74	0.311
Bi (ng/g)	0.004	20	0.006	0.002	420	0.003	20	0.003	0.001	400	0.397
Ca (μg/g)	109.7	20	89.12	72	394.5	133.9	20	165.8	74.5	425.5	0.341
Cd (ng/g)	0.01	20	0.01	0.008	432.5	0.009	20	0.006	0.007	387.5	0.275
Co (ng/g)	0.01	20	0.01	0.006	376.5	0.011	20	0.011	0.007	443.5	0.183
Cr (ng/g)	0.08	19	0.08	0.03	373	0.05	20	0.04	0.038	407	0.425
Cu (μg/g)	4.38	20	0.90	4.07	491.5	3.60	20	1.01	3.735	328	0.013 *
Fe (μg/g)	86.3	20	44.56	71	414.5	102.4	20	86.68	61	405.5	0.454
K (μg/g)	2612	20	335.3	2401	488	2359	20	318.7	2401	332	0.017 *
Li μg/g)	0.02	15	0.03	0.01	249	0.17	15	0.36	0.007	216	0.252
Mg (μg/g)	113.4	20	16.4	109	499	100.9	20	15.22	100.5	321	0.007 *
Mn (ng/g)	0.37	20	0.17	0.32	448	0.36	20	0.25	0.23	372	0.155
Mo (ng/g)	0.04	20	0.02	0.04	468	0.04	20	0.01	0.03	352	0.059
Na (μg/g)	1548	20	310.2	1613	437	1469	20	284.8	1414	383	0.238
Ni (ng/g)	0.13	20	0.12	0.07	465	0.09	20	0.10	0.07	355	0.069
Pb (ng/g)	0.06	20	0.12	0.02	433.5	0.05	20	0.09	0.01	386.5	0.266
Rb (μg/g)	2.25	20	0.51	2.18	425.5	2.21	20	0.67	2.12	394.5	0.341
Sr (ng/g)	0.08	20	0.09	0.04	426	0.08	20	0.13	0.04	394	0.339
U (ng/g)	0.0004	20	0.0002	0.0003	445.5	0.0003	20	0.0002	0.0002	374.5	0.172
V (ng/g)	0.04	20	0.11	0.006	478.5	0.005	20	0.003	0.0045	341.5	0.030 *
Zn (μg/g)	16.27	20	11.18	14	451.5	14.75	20	8.47	12	368.5	0.132

Prefrontal brain samples were obtained from the human postmortem brain tissue collection at the Human Brain Collection Core, National Institute of Mental Health (NIMH). Metals were determined as described in Materials and Methods. * Significantly lower concentrations compared to control.

**Table 5:** Spearman’s rank correlations coefficient between elemental concentrations in PFC of BD patients and control subjects with age.

Element	Control	Bipolar Disorder
Al (μg/g)	−0.277	−0.074
B (ng/g)	−0.176	0.160
Ba (ng/g)	−0.056	0.482
Be (μg/g)	0.106	−0.027
Bi (ng/g)	−0.520	-0.117
Ca (μg/g)	−0.113	0.241
Cd (ng/g)	0.458	−0.128
Co (ng/g)	−0.198	−0.163
Cr (ng/g)	−0.221	0.348
Cu (μg/g)	−0.208	0.032
Fe (μg/g)	−0.084	−0.135
K (μg/g)	−0.374	0.329
Li (μg/g)	−0.138	0.022
Mg (μg/g)	−0.101	0.338
Mn (ng/g)	0.089	0.067
Mo (ng/g)	−0.195	0.039
Na (μg/g)	0.256	−0.030
Ni (ng/g)	0.111	−0.118
Pb (ng/g)	−0.029	−0.037
Rb (μg/g)	0.108	0.391
Sr (ng/g)	−0.031	0.205
U (ng/g)	−0.061	0.313
V (ng/g)	−0.106	0.174
Zn (μg/g)	0.048	0.460

Spearman’s rank correlations coefficient between brain element concentrations in control and BD samples and age before and after treatment were calculated using GraphPad Prism v 7.03.

**Table 6:** Inter-elemental Spearman’s Rank Significant Correlations in sera samples of bipolar patients before and after treatment.

Before Treatment		After Treatment	
Element	Correlated with	Element	Correlated with
Fe	K, Li, Mg, **Na**, PO_4_, Rb	Fe	K, Mn, **Na**, Rb, Zn
K	Li, Mg, **Na**, PO_4_, Rb	K	Mg, **Na**, **Ni**, Rb, Zn
Li	Mg, **Na**, Rb, Zn	Li	**SO_4_**
Mg	**Na**, PO_4_, Rb	Na	**Rb**
Ca	PO_4_, SO_4_	Co	Zn
Na	**Rb**, **Zn**	Cu	**Li**
Ba	Mn	Mn	Zn
Co	Ni	B	**Mn**
Cr	V		
Cu	SO_4_		
Mn	Zn		
Ni	Pb		

The Inter-element Spearman’s Rank Significant Correlations in sera samples of bipolar patients before and after treatment are depicted. Positive correlations are presented in non-boldface and negative correlations in boldface. Detailed data of the correlations and statistical significance are presented in Tables S1 and S2.

**Table 7:** Inter-elemental Spearman’s Rank significant correlations in PFC of control and bipolar disorder subjects.

Control		Bipolar Disorder	
Element	Correlated with	Element	Correlated with
Al	B, Ba, Ca, Co, Cr, Li, Ni, Pb, Sr, U, V	Al	V
Ca	Cd, Co, Cr, Ni, Pb, Sr, U, V, Zn	Ca	Ni, Pb, Sr, U
Co	Cr, Fe, Mn, Ni, Pb, Sr, U, V, Zn	Co	Mn
Ba	Co, Cr, Fe, Li, Mn, Sr, U, V	Ba	Ca, Sr, U
Cr	Li, Ni, Pb, Sr, U, V, Zn	Cr	V
B	Ca, Ni, Pb, Sr, U	B	Bi, Mg
Ni	Pb, Sr, U, V, Zn	Ni	Pb, Sr
Li	Ni, Sr, U, V	Li	**Pb**
Cu	Mg, Na, Rb	Cu	Mg, **U**, **V**
Mg	Na, Ni, Rb	Mg	Mn, Rb
Pb	Sr, U, V	Pb	Sr, V
Fe	Mn, Zn	Fe	K, **Li**, Mn
Cd	Ni, Sr	Cd	Mn
Sr	U, V	Sr	U
K	Rb	K	Mg, Mn, **Na**, Rb
Mn	Zn	Mn	Zn
Be	Pb	U	V
U	V	Bi	**Li**, Mo

The Inter-element Spearman’s Rank Significant Correlations in PFC of Control and Bipolar Disorder Subjects are depicted. Positive correlations are presented in non-boldface and negative correlations in boldface. Detailed data of the correlations and statistical significance are presented in Tables S1 and S2 (see Supplementary Materials).

## Data Availability

Not applicable.

## Supplementary Materials

The following supporting information can be downloaded at: https://www.mdpi.com/article/10.3390/ijms232214362/s1.

## Abbreviations

BD	Bipolar disease
PFC	Prefrontal cortex
ICP-MS	Inductively coupled plasma mass spectrometry
r_s_	Spearman’s correlation coefficient
Na^+^, K^+^-ATPase	Sodium, Potassium-activated Adenosine Triphosphatase
