# Supplementary material for: Chemical Element Profiling in the Sera and Brain of Bipolar Disorders Patients and Healthy Controls
Source: Int J Mol Sci. 2022 Nov 18;23(22):14362. doi: 10.3390/ijms232214362 (PMC9692593; doi:10.3390/ijms232214362)
Supplement: Supplementary file 1 [file ijms-23-14362-s001.zip › ijms-1942791-supplementary.pdf]

Table S1. Spearman's rank correlations coefficient between elemental concentrations in sera samples before treatment

	**B (ppb)**	**Ba (ppb)**	**Ca (ppm)**	**Co (ppb)**	**Cr (ppb)**	**Cu (ppm)**	**Fe (ppm)**	**K (ppm)**	**Li (ppm)**	**Mg (ppm)**	**Mn (ppb)**	**Na (%)**	**Ni (ppb)**	**Pb (ppb)**	**PO_4 (ppm)**	**Rb (ppm)**	**SO_4 (%)**	**Sr (ppb)**	**V (ppb)**	**Zn (ppm)**
**B**	1.000																			
**Ba**	0.266	1.000																		
**Ca**	0.526	0.053	1.000																	
**Co**	-0.179	0.141	-0.384	1.000																
**Cr**	0.278	0.569	0.087	0.240	1.000															
**Cu**	-0.060	-0.153	0.471	-0.404	-0.490	1.000														
**Fe**	0.009	0.366	0.293	-0.130	0.046	0.364	1.000													
**K**	-0.005	0.280	0.385	-0.356	0.087	0.443	***0.947***	1.000												
**Li**	0.192	0.507	0.387	-0.151	0.099	0.406	**0.837**	**0.814**	1.000											
**Mg**	0.212	0.102	0.464	-0.453	-0.125	0.579	**0.788**	**0.859**	*0.636*	1.000										
**Mn**	0.210	*0.717*	-0.322	0.238	0.034	-0.072	0.320	0.126	0.372	0.146	1.000									
**Na**	0.157	-0.420	0.061	-0.089	-0.028	-0.211	**-0.777**	*-0.724*	*-0.699*	*-0.667*	-0.488	1.000								
**Ni**	0.070	0.040	-0.296	*0.626*	0.145	-0.319	-0.317	-0.449	-0.211	-0.378	0.134	-0.086	1.000							
**Pb**	-0.089	-0.028	-0.299	0.400	0.108	-0.161	-0.196	-0.187	-0.042	-0.057	0.047	-0.386	**0.760**	1.000						
**PO_4**	0.391	0.050	*0.668*	-0.237	-0.073	0.373	*0.718*	*0.674*	0.549	*0.682*	0.023	-0.232	-0.504	-0.547	1.000					
**Rb**	0.119	0.305	0.323	-0.515	0.018	0.420	***0.870***	***0.940***	**0.807**	***0.866***	0.224	*-0.728*	-0.367	-0.098	0.556	1.000				
**SO_4**	0.317	-0.044	***0.923***	-0.361	0.023	*0.615*	0.207	0.323	0.213	0.434	-0.393	0.141	-0.382	-0.359	0.584	0.203	1.000			
**Sr**	0.582	0.201	0.403	0.241	-0.055	-0.138	0.018	-0.146	0.256	-0.120	0.233	0.148	0.075	-0.173	0.400	-0.160	0.170	1.000		
**V**	-0.296	0.233	-0.286	0.567	*0.666*	-0.225	0.065	0.069	0.081	-0.183	-0.051	-0.235	0.372	0.442	-0.319	-0.074	-0.213	-0.374	1.000	
**Zn**	0.096	0.585	0.055	0.169	-0.075	0.173	0.497	0.381	*0.704*	0.335	*0.688*	*-0.631*	-0.112	0.103	0.251	0.374	-0.065	0.460	-0.063	1.000

Spearman's rank correlations coefficient between elemental concentrations in sera samples before treatment were calculated using Graphpad Prism v 7.03. ppb=μg/L and ppm=mg/L. *p* value: $p < 0.05$ (Italics, blue), $p < 0.01$ (bold, red), $p < 0.001$ (bold, Italics & green)

Table S2. Spearman's rank correlations coefficient between elemental concentrations in sera samples after treatment

	B (ppb)	Ba (ppb)	Ca (ppm)	Co (ppb)	Cr (ppb)	Cu (ppm)	Fe (ppm)	K (ppm)	Li (ppm)	Mg (ppm)	Mn (ppb)	Na (%)	Ni (ppb)	Pb (ppb)	PO_4 (ppm)	Rb (ppm)	SO_4 (%)	Sr (ppb)	V (ppb)	Zn (ppm)
**B**	1.000																			
**Ba**	0.205	1.000																		
**Ca**	0.097	-0.188	1.000																	
**Co**	-0.434	0.117	0.233	1.000																
**Cr**	-0.533	0.352	-0.483	0.508	1.000															
**Cu**	-0.033	-0.122	0.403	-0.218	-0.058	1.000														
**Fe**	-0.450	-0.005	-0.082	0.110	0.237	-0.392	1.000													
**K**	-0.532	-0.083	0.169	0.010	0.178	0.115	**0.763**	1.000												
**Li**	0.460	-0.025	-0.183	-0.105	-0.274	*-0.651*	0.160	-0.460	1.000											
**Mg**	-0.303	-0.012	0.385	0.121	0.021	-0.075	0.508	*0.669*	-0.238	1.000										
**Mn**	*-0.659*	-0.016	-0.141	0.612	0.525	-0.464	*0.708*	0.593	-0.134	0.460	1.000									
**Na**	0.200	0.103	0.194	0.319	-0.106	0.019	**-0.768**	*-0.689*	-0.037	-0.152	-0.312	1.000								
**Ni**	0.501	0.206	0.303	-0.031	-0.373	0.121	-0.547	*-0.621*	0.244	-0.362	-0.588	0.354	1.000							
**Pb**	-0.370	0.293	0.042	0.112	0.363	0.249	0.283	0.408	-0.372	0.026	0.122	-0.474	0.193	1.000						
**PO_4**	-0.257	0.393	0.037	0.096	-0.041	-0.341	0.336	0.391	-0.187	0.587	0.437	0.087	-0.308	-0.116	1.000					
**Rb**	-0.400	-0.023	0.286	-0.074	-0.073	0.012	**0.769**	***0.943***	-0.311	0.026	0.495	*-0.657*	-0.454	0.379	0.524	1.000				
**SO_4**	-0.214	0.131	0.093	0.012	0.032	0.423	-0.157	0.336	**-0.788**	-0.091	0.082	-0.105	0.021	0.473	0.175	0.303	1.000			
**Sr**	0.413	-0.351	-0.187	-0.440	-0.360	-0.419	0.264	0.100	0.474	0.009	-0.037	-0.363	-0.322	-0.339	-0.155	0.137	-0.318	1.000		
**V**	-0.553	0.095	-0.258	-0.136	0.433	0.329	0.000	0.221	-0.494	0.073	-0.070	-0.107	-0.119	0.603	-0.019	0.113	0.234	-0.351	1.000	
**Zn**	-0.602	0.144	0.105	*0.665*	0.546	-0.249	**0.760**	*0.633*	-0.114	0.516	***0.889***	-0.364	-0.461	0.265	0.360	0.553	-0.012	-0.210	-0.072	1.000

Spearman's rank correlations coefficient between elemental concentrations in sera samples after treatment were calculated using Graphpad Prism v 7.03. ppb=µg/L and ppm=mg/L. *p* value: $p < 0.05$ (Italics, blue), $p < 0.01$ (bold, red), $p < 0.001$ (bold, Italics & green)

Table S3. Spearman's rank correlations coefficient between elemental concentrations in PFC of Control subjects

	**Al (ppm)**	**B (ppb)**	**Ba (ppb)**	**Be (ppm)**	**Bi (ppb)**	**Ca (ppm)**	**Cd (ppb)**	**Co (ppb)**	**Cr (ppb)**	**Cu (ppm)**	**Fe (ppm)**	**K (ppm)**	**Li (ppm)**	**Mg (ppm)**	**Mn (ppb)**	**Mo (ppb)**	**Na (ppm)**	**Ni (ppb)**	**Pb (ppb)**	**Rb (ppm)**	**Sr (ppb)**	**U (ppb)**	**V (ppb)**	**Zn (ppm)**
**Al**	1.000																							
**B**	*0.502*	1.000																						
**Ba**	*0.492*	0.254	1.000																					
**Be**	-0.421	0.053	0.092	1.000																				
**Bi**	0.377	0.049	0.310	0.102	1.000																			
**Ca**	**0.640**	*0.471*	0.222	0.381	0.091	1.000																		
**Cd**	0.286	0.158	0.040	-0.225	-0.403	*0.516*	1.000																	
**Co**	**0.638**	0.234	**0.574**	0.327	0.302	***0.706***	0.296	1.000																
**Cr**	***0.788***	0.439	*0.489*	-0.026	0.340	**0.659**	0.249	***0.707***	1.000															
**Cu**	0.264	0.092	0.010	0.531	0.241	0.273	0.074	0.104	0.218	1.000														
**Fe**	0.081	-0.089	*0.561*	0.333	0.165	0.150	-0.016	**0.614**	0.425	0.047	1.000													
**K**	-0.162	-0.214	-0.278	0.145	0.079	0.021	-0.162	0.010	0.047	0.283	0.206	1.000												
**Li**	**0.767**	0.318	***0.891***	0.085	0.483	0.236	0.061	0.487	*0.609*	0.452	0.488	-0.372	1.000											
**Mg**	0.351	0.436	0.144	0.317	0.183	0.268	0.008	0.252	0.435	***0.692***	0.261	0.353	0.327	1.000										
**Mn**	0.038	-0.093	*0.477*	0.295	0.001	0.271	0.281	**0.589**	0.359	0.027	***0.908***	0.169	0.322	0.205	1.000									
**Mo**	0.063	-0.107	-0.304	0.484	0.296	0.142	0.048	0.100	0.337	0.427	0.054	0.246	0.010	0.347	0.042	1.000								
**Na**	0.136	0.066	-0.137	-0.082	-0.058	0.178	0.298	-0.213	0.142	*0.542*	-0.014	0.258	0.181	*0.456*	0.110	0.242	1.000							
**Ni**	**0.673**	*0.454*	0.311	0.482	0.182	***0.786***	*0.54*	**0.618**	***0.715***	0.380	0.193	-0.114	*0.556*	*0.453*	0.328	0.389	0.325	1.000						
**Pb**	*0.457*	*0.491*	0.380	**0.791**	0.179	**0.643**	0.336	*0.503*	*0.554*	0.333	0.046	-0.111	0.165	0.370	0.116	0.171	-0.043	**0.63**	1.000					
**Rb**	-0.203	-0.331	0.016	0.357	-0.070	-0.062	-0.005	0.090	-0.0193	*0.47*	0.206	*0.534*	-0.127	*0.481*	0.182	0.376	0.214	0.135	0.090	1.000				
**Sr**	***0.746***	*0.489*	*0.459*	0.319	0.254	***0.883***	*0.53*	***0.695***	**0.67**	0.175	0.132	-0.251	*0.568*	0.181	0.216	0.100	0.123	***0.8***	***0.693***	-0.190	1.000			
**U**	***0.877***	*0.474*	**0.609**	0.223	0.389	***0.746***	0.358	**0.649**	***0.715***	0.309	0.176	-0.252	**0.763**	0.308	0.196	0.049	0.208	***0.77***	**0.649**	-0.069	***0.896***	1.000		
**V**	***0.878***	0.430	**0.570**	0.019	0.407	**0.637**	0.359	**0.662**	***0.8***	0.224	0.297	-0.190	***0.781***	0.352	0.350	0.213	0.278	***0.74***	*0.49*	-0.100	***0.767***	***0.855***	1.000	
**Zn**	0.189	-0.069	0.365	0.484	0.301	*0.467*	0.100	*0.501*	*0.466*	0.173	*0.532*	0.382	0.169	0.355	**0.603**	0.176	0.227	*0.49*	0.432	0.367	0.437	0.410	0.368	1.000

Spearman's rank correlations coefficient between the elemental concentrations in PFC of Control subjects was performed using Graphpad Prism v7.03. ppb=ng/g and ppm=μg/g. *p* value: $p < 0.05$ (Italics, blue), $p < 0.01$ (bold, red), $p < 0.001$ (bold, Italics & green)

Table S4. Spearman's rank correlations coefficient between elemental concentrations in PFC of Bipolar Disorder subjects

	**Al (ppm)**	**B (ppb)**	**Ba (ppb)**	**Be (ppm)**	**Bi (ppb)**	**Ca (ppm)**	**Cd (ppb)**	**Co (ppb)**	**Cr (ppb)**	**Cu (ppm)**	**Fe (ppm)**	**K (ppm)**	**Li (ppm)**	**Mg (ppm)**	**Mn (ppb)**	**Mo (ppb)**	**Na (ppm)**	**Ni (ppb)**	**Pb (ppb)**	**Rb (ppm)**	**Sr (ppb)**	**U (ppb)**	**V (ppb)**	**Zn (ppm)**
**Al**	1.000																							
**B**	-0.044	1.000																						
**Ba**	0.057	0.377	1.000																					
**Be**	0.383	0.114	0.237	1.000																				
**Bi**	0.219	*0.526*	-0.015	0.534	1.000																			
**Ca**	0.274	0.077	*0.536*	0.420	-0.129	1.000																		
**Cd**	-0.196	0.286	-0.107	0.385	0.184	-0.387	1.000																	
**Co**	-0.194	0.344	0.071	-0.168	0.327	-0.031	0.161	1.000																
**Cr**	0.032	0.048	0.375	0.164	-0.203	0.291	0.261	0.220	1.000															
**Cu**	-0.224	0.277	0.020	0.383	0.095	-0.235	0.354	0.006	-0.205	1.000														
**Fe**	0.204	0.251	-0.040	-0.660	0.077	-0.228	0.195	0.382	0.006	0.142	1.000													
**K**	0.152	0.304	0.099	0.073	0.360	-0.340	0.140	0.413	-0.029	0.226	*0.511*	1.000												
**Li**	-0.416	-0.525	-0.259	0.059	*-0.588*	-0.312	-0.123	-0.199	-0.236	-0.180	*-0.596*	-0.151	1.000											
**Mg**	-0.140	**0.600**	0.278	0.201	0.270	-0.247	0.307	0.203	0.029	***0.711***	0.402	*0.554*	-0.456	1.000										
**Mn**	-0.087	0.400	-0.016	-0.219	0.211	-0.424	**0.607**	*0.506*	0.233	0.386	***0.731***	**0.654**	-0.384	**0.585**	1.000									
**Mo**	0.185	0.412	0.100	0.249	*0.532*	0.163	-0.059	0.089	-0.218	0.048	-0.005	0.085	-0.245	0.169	0.057	1.000								
**Na**	-0.233	-0.082	-0.196	0.256	-0.015	0.029	0.405	-0.100	0.193	0.316	-0.337	*-0.467*	0.009	-0.009	-0.071	0.085	1.000							
**Ni**	0.206	-0.041	0.128	0.230	0.052	**0.562**	-0.205	0.212	0.229	-0.108	0.068	-0.266	-0.418	-0.045	-0.136	0.346	0.142	1.000						
**Pb**	0.218	0.141	0.260	-0.193	0.172	*0.454*	-0.178	0.373	0.396	-0.327	0.098	-0.112	*-0.591*	-0.097	0.002	0.210	0.105	***0.721***	1.000					
**Rb**	0.190	0.259	0.033	0.420	0.295	-0.144	-0.020	0.111	-0.015	0.309	0.205	***0.704***	-0.204	**0.624**	0.308	0.157	-0.296	-0.121	-0.255	1.000				
**Sr**	0.143	0.183	*0.517*	0.365	0.024	***0.881***	-0.317	0.297	0.444	-0.312	-0.274	-0.250	-0.293	-0.223	-0.282	0.270	0.078	**0.623**	**0.670**	-0.135	1.000			
**U**	0.292	0.082	*0.543*	0.219	0.064	**0.666**	-0.211	0.241	0.373	*-0.475*	-0.149	0.141	-0.125	-0.219	-0.152	0.102	-0.286	0.309	0.423	0.175	***0.748***	1.000		
**V**	**0.589**	-0.017	0.213	0.304	0.075	0.297	0.068	0.115	***0.684***	*-0.537*	0.034	0.075	-0.239	-0.170	0.028	0.095	-0.009	0.325	*0.489*	0.129	0.430	**0.576**	1.000	
**Zn**	-0.032	0.160	0.245	-0.065	-0.183	0.200	-0.032	0.208	0.251	0.355	0.335	0.361	-0.217	0.435	*0.489*	0.278	0.082	0.269	0.309	0.228	0.266	0.109	0.039	1.000

Spearman's rank correlations coefficient between elemental concentrations in PFC of Bipolar Disorder subjects was performed using Graphpad Prism v7.03. ppb=ng/g and ppm=μg/g. *p* value: $p < 0.05$ (Italics, blue), $p < 0.01$ (bold, red), $p < 0.001$ (bold, Italics & green)
